# Novel Natural-based Biomolecules Discovery for Tackling Chronic Diseases

**DOI:** 10.3390/biom10121674

**Published:** 2020-12-15

**Authors:** Hang Fai Kwok

**Affiliations:** 1 Institute of Translational Medicine, Faculty of Health Sciences, University of Macau, Avenida de Universidade, Macau SAR, China; hfkwok@um.edu.mo; 2 Cancer Center, Faculty of Health Sciences, University of Macau, Avenida de Universidade, Macau SAR, China

In the last decade, natural-derived/-based biomolecules have continuously played an important role in novel drug discovery (as a prototype drug template) for potential chronic disease treatment. Many recent research studies have demonstrated that the development of natural peptide/protein-based, toxin-based, and antibody-based drugs can significantly improve the biomedical efficiency of disease-specific therapy.

The focus of this Special Issue of Biomolecules includes eleven papers: ten original research articles and one communication article from nine different countries/regions dealing with a broad range of the discovery and development of the natural biomolecules as potential medical therapy for tackling chronic diseases (e.g., cancer, diabetes, cardiovascular diseases, rheumatoid arthritis, and pain treatment)

Four cancer-related research articles by Wang Q. et al. [1], Miao Y. et al. [2], Martínez-García D. et al. [3], Thangaraj K. et al. [4], and Lai Y. et al. [5] demonstrate that several natural-based compounds/proteins could effectively influence the cancer formation and progression. These study findings unveil the relationship of the SUMO pathway and DAPK1 protein degradation, demonstrate the target modifications of novel protease could effectively and efficiently alter its anticancer bioactivity, study the survivin levels through potent STAT3 Inhibition in lung cancer, investigate the cell cycle arrest and mitochondria-mediated intrinsic apoptosis in colorectal carcinoma, and develop a Fucoidan-based drug delivery system by using hydrophilic anticancer polysaccharides to simultaneously deliver hydrophobic anticancer drugs, respectively.

For potential diabetes therapy, the fascinating study by Lee D. et al. [6] evaluates two isoflavonoids and a nucleoside which were isolated from the roots of *Astragalus membranaceus*. These bioactive compounds can improve insulin secretion in β-cells, representing the first step towards the development of potent antidiabetic drugs. Besides, the paper by Martínez-Navarro I. et al. [7] concludes that the anionic lipid environment and degree of solvation are critical conditions for the stability of segments with the propensity to form β-sheet structures. This situation will eventually affect the structural characteristics and stability of IAPP within insulin granules, thus modifying the insulin secretion.

Two articles deals with inflammatory-related diseases. The Lee J. S. et al. [8] study speculates on two characterized compounds, petasitesin A (1) and cimicifugic acid D (3), which are worthy of further pharmacological evaluation for their potential as anti-inflammatory drugs. In addition, Celiksoy V. et al. [9] aimed to examine punicalagin in combination with Zn (II), and demonstrate that this novel combination promotes anti-inflammatory and fibroblast responses to aid oral healing.

The article by Szűcs E. et al. [10] explored the biological effect of novel opioid peptide analogs incorporating L-kynurenine (L-kyn) and kynurenic acid (kyna) in place of native amino acids. This novel oligopeptide exhibits a strong antinociceptive effect after i.c.v. and s.c. administrations in in vivo tests, according to good stability in human plasma which has a potential for tackling pain syndromes. 

Finally, the short communication paper by Maheshwari G. et al. [11] tested the hypothesis that monomethyl branched-chain fatty acids (BCFAs) and a lipid extract of Conidiobolus heterosporus (CHLE) can activate the nuclear transcription factor peroxisome proliferator-activated receptor alpha (PPARalpha). In conclusion, they showed that the monomethyl BCFA isopalmitic acid (IPA) IPA is a potent PPARalpha activator. CHLE activates PPARalpha-dependent gene expression in Fao cells, an effect that is possibly mediated by IPA.

Overall, this Special Issue describes important findings related to natural-derived/-based biomolecules for potential chronic diseases treatment by dysregulating several biological pathways/receptors. It also highlights the most recent progress on the knowledge and the clinical and pharmacological applications related to the most relevant areas of healthcare.
